# Research on efficient numerical simulation method for integration fracking with production in shale oil reservoir with multi-source data

**DOI:** 10.1038/s41598-024-81896-9

**Published:** 2024-12-23

**Authors:** Jie Zhan, Xifeng Ding, Hai Liu, Kongjie Wang, Zhipeng Wang, Wenting Guo, Ren-Shi Nie, Xianlin Ma, Zhenzihao Zhang

**Affiliations:** 1 School of Petroleum Engineering, Xi’an Shiyou University, Xi’an, 710065 China; 2 Changqing Downhole Technology Company, CNPC Chuanqing Drilling Engineering Company Limited, Xi’an, 710018 China; 3 State Key Laboratory of Petroleum Resources and Prospecting, China University of Petroleum (Beijing), Beijing, 102249 China; 4 State Key Laboratory of Oil and Gas Reservoir Geology and Exploitation, Southwest Petroleum University, Chengdu, 610500 Sichuan China

**Keywords:** Numerical simulation, The DG model, Multi-stage history matching, Dynamic evolution, Shale oil, Fossil fuels, Energy infrastructure, Geodynamics, Geology, Fluid dynamics

## Abstract

Horizontal well hydraulic fracturing technology has significantly enhanced the productivity of shale reservoirs. However, our understanding of the expansion patterns within the complex fracture network and fluid seepage mechanisms under field conditions remains inadequate. Here, this work develops a dynamic geomechanical (DG) model to simulate the complete sequence of operations in hydraulic fracturing. This study utilizes a construction procedure that closely mirrors field practices to establish the DG model. Furthermore, the numerical simulation results of the DG model are calibrated with field data. This work adopts a numerical simulation method that integrates unsteady seepage model for multi-stage fractured horizontal wells with the dilation-recompaction model to develop the DG model. It systematically constructs the geological model of the shale reservoir by utilizing segmented logging data and by segmenting production data. The time series evolution system is developed through an iterative process involving discrete time steps. Results show that the DG model can perform history matching on a multi-stage basis, enabling comprehensive and detailed analysis of the entire reservoir. This process effectively replicates the distribution relationship between each reconstruction zone and the overall productivity. Furthermore, the DG model is capable of accurately simulating the dynamic process of injected high-pressure fluids into the reservoir to fracture the rock and the dynamic evolution law of reservoir properties. Hydraulic fracturing creates a fracture zone that centers on the well’s border and spreads outward radially. The injection volume and failure pressure are significantly correlated with the scale of shale reservoir reconstruction. Following the injection of 790.5 m³ of fracturing fluid in the first stage, the fracture half-length can reach around 148 m, essentially fulfilling the design specifications. Permeability can reach up to 86 mD at this moment, and it can even be maintained at the level of 46 mD during production. In conclusion, the DG model broadens the focus of study on the development of shale reservoirs and lays the groundwork for improving productivity and optimizing hydraulic fracturing design.

## Introduction

The low porosity and low permeability characteristics of shale reservoirs result in low development efficiency and rapid production decline^1–3^. Therefore, the implementation of effective stimulation techniques such as hydraulic fracturing and horizontal drilling has become essential^4–7^. Developing shale reservoirs typically involves a prolonged, low-productivity process due to their unique geologic characteristics. The application of hydraulic fracturing and other techniques is believed to enhance the flow paths of oil and gas by creating new channels^8^. In the reservoir near the wellbore, hydraulic fracturing commonly results in the formation of a complex fracture network. This fracture network significantly enhances the area’s permeability, thereby improving the performance of production well^5^. Numerical simulation technology is utilized to meticulously examine the geometry and connectivity of complex fractures, providing a detailed understanding of the well production dynamics. However, the characterization of the expansion patterns of the complex fracture network and fluid seepage mechanisms under field conditions is challenging due to intricate seepage characteristics, the requirement for high precision in simulation grids division, and discontinuous fluid parameters at the fracture interface. It is challenging to appropriately characterize fluid flow within the complete fracture network of shale reservoirs using a basic analytical model^9,10^.

When defining the fracture and the matrix, the standard equivalent medium model requires a substantial amount of meshing work, which leads to significant computing complexity and low efficiency^11,12^. Although it has some limitations concerning geometric scale characteristics and connectivity, especially for large-scale fractures in reservoirs, the dual porosity model accounts for the fluid flow difference between the matrix and fracture systems^13^. Even though the discretization based on unstructured grids leads to complicated iterative procedures and poor convergence, the introduction of the discrete fracture model simplifies the understanding of the geometric shape, size, and spatial distribution of fractures. However, complicated hydraulic fracture shape in shale reservoirs makes it hard to use computer tools efficiently when the grid is divided in an irregular way^14–17^. Furthermore, the embedded fracture model represents the fluid exchange between the matrix grid and the fracture by treating the fracture as a well source in the matrix grid and using a mathematical equation. Although the model is effective in meshing, it is not able to accurately describe the process of multiphase fluid exchange between the matrix grid and the fracture^18–22^.

The geomechanical model of dilation-recompaction was first introduced by Beattle et al.^23^. The Cold Lake heavy oil reservoir’s oil sand dilatation and recompaction during cyclic steam stimulation (CSS) were deeply examined, and a satisfactory match of pressure was achieved during the production and steam injection stages. One of the primary benefits of replicating the hydraulic fracturing process with the dilation-recompaction approach is that the geometric shape and length scale of the fracture do not need to be preset^24^. Fracture matching is accomplished by modifying the model control parameters. The findings indicate that the degree of fracture development predicted by the model yield is fair and that it matches substantially with the actual field data. Moreover, during the water flooding process in low-permeability reservoirs, numerical simulation based on the dilation-recompaction model can effectively match historical production dynamics and quantitatively describe the fluid-solid coupling behavior of water flooding^25–28^. This study not only offers theoretical support for numerical simulation technology in reservoir hydraulic fracturing but also presents a robust method for simulating fluid-rock interaction under complex geological conditions.

This work employs the numerical simulation method that couples the unsteady seepage model of multi-stage fractured horizontal wells and the dilation-recompaction model to enhance comprehension and optimization of this study. Using the field case of multi-stage fractured horizontal well in the Chang 7 reservoir of Changqing Oilfield verifies the practicality of the DG model. This paper conducts a deep investigation of the hydraulic fracturing production process in shale reservoirs by coupling the geomechanical model of dilation-recompaction with the unsteady seepage model. Hydraulic fracturing operation leads to the dynamic evolution of reservoir porosity and permeability. Porosity is pressure-dependent and alters in response to the injection of high-pressure fluid. Permeability is a function of porosity and serves as a key parameter for characterizing the dynamic fracture propagation within shale reservoirs. Through iterative calculations across time steps, the evolution of fracture growth during hydraulic fracturing can be validly simulated. The geological model of the shale reservoir is established, and the multi-stage history matching is performed by using an operation procedure essentially aligned with field construction practices. The comprehensive history matching of the entire reservoir is achieved to finish the deep restoration of the relationship between each reconstruction zone and the overall productivity distribution. This work enables a profound analysis and investigation of the fracturing production process of the shale reservoir, providing a scientific basis for optimizing fracturing design and improving productivity.

## Methodology

### System layout

**Fig. 1:**
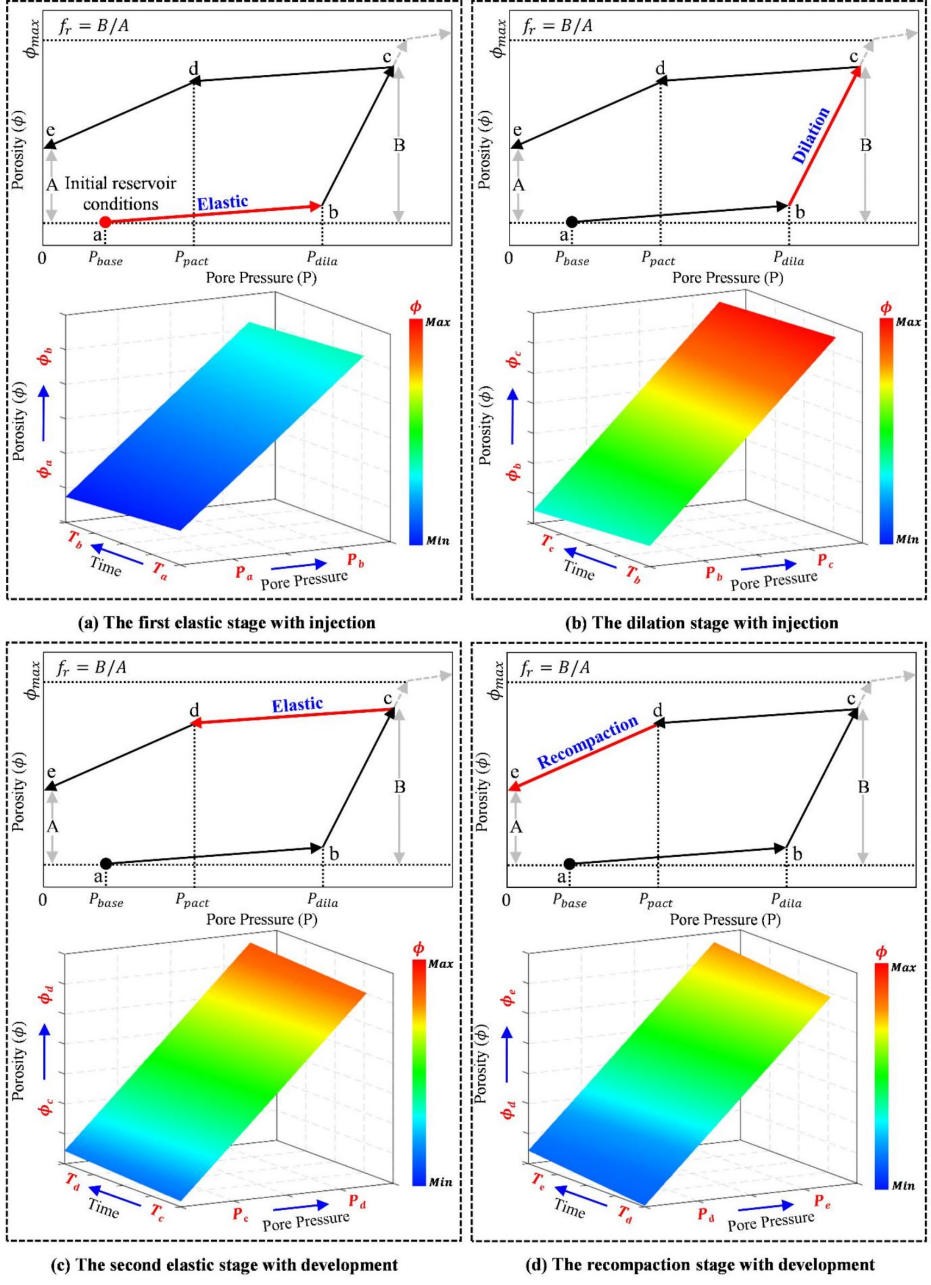
The dynamic properties evolution of the DG model^26^.

This work applies the dilation-recompaction model to characterize the multi-stage hydraulic fracturing process in horizontal wells within shale reservoirs. Figure 1 illustrates the complete periodic variation process of pores generated by the DG model under various pressure conditions.

The DG model demonstrates the dynamic evolution of porosity in response to the pressure varies, highlighting the alterations in the rock pore structure at different stages. The injection of fracturing fluid causes a gradual increase in the formation pressure ($\:{P}_{\text{b}\text{a}\text{s}\text{e}}$) from its initial value. Elastic deformation results in an increase in porosity from point *a* to point *b*. As fracturing fluid is injected, the pressure gradually builds-up. As soon as the pressure reaches the expansion starting pressure ($\:{P}_{\text{d}\text{i}\text{l}\text{a}}$), the porosity rises following line *bc*. The porosity decreases along the elastic compaction line *cd* if the pressure starts to drop at a particular position on the dilation line. When the pressure is further reduced below the recompaction critical pressure ($\:{P}_{\text{p}\text{a}\text{c}\text{t}}$), the recompaction process begins, and the slope of the recompaction line is determined by the residual dilation coefficient ($\:{f}_{\text{r}}$). A new cycle of dilation and recompaction begins when the pressure starts to increase from a specific point on the recompaction curve.

Regardless of the influence of temperature on the pore volume of the reservoir, the relationship between porosity and permeability of the reservoir with pressure will be expressed in the form of a function^23,29^, where the function of porosity is

1 $$\:\varphi\:={\varphi\:}_{ref}{e}^{\left[{C}_{p}\left(P-{P}_{ref}\right)\right]}.$$

where $\:{P}_{\text{r}\text{e}\text{f}}$ represents the reference pressure, $\:{\varphi\:}_{\text{r}\text{e}\text{f}}$ signifies the porosity under $\:{P}_{\text{r}\text{e}\text{f}}$, and $\:{C}_{\text{p}}$ denotes the pore volume compression coefficient. Each curve branch in the model is associated with a set of data for these three variables. The change in permeability is a function of porosity, so the equation for permeability is

2 $$\:K={K}_{0}{e}^{\left[{K}_{mul}\left(\varphi\:-{\varphi\:}_{ref}\right)/\left(1-{\varphi\:}_{ref}\right)\right]}.$$

where $\:{K}_{0}$ is the initial permeability, $\:{K}_{\text{m}\text{u}\text{l}}$ is the permeability multiple, which is used to adjust the permeability to meet the needs of history matching.

**Fig. 2:**
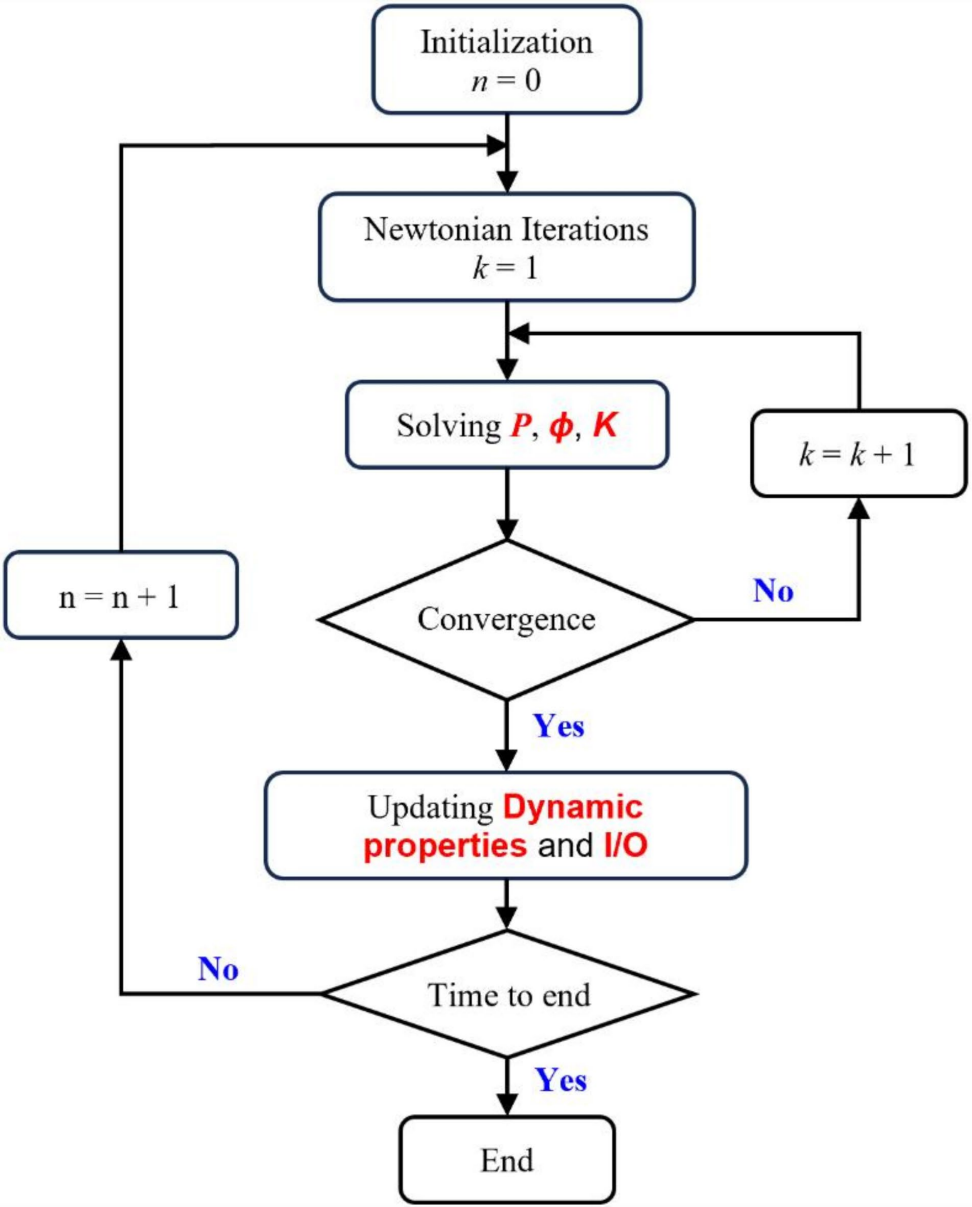
Numerical simulation iteration method flowchart^30^.

Figure 2 displays numerical simulation and the dynamic geomechanical properties iterative calculation procedure based on the DG model. The initial data must be initialized before the numerical simulation is performed. The *n* represents the number of timestep iterations and the *k* represents the number of newtonian iterations. The reservoir pressure, porosity and permeability are solved by iterative calculation. Data on computed rock and fluid properties in every phase are applied to derive new porosity and permeability. Once the convergence condition is satisfied through iterative solving, the dynamic properties are updated, and the input or output for the next timestep are determined. The iterative algorithm is reiterated until the outcome satisfies the pre-specified convergence condition. The iteration process is governed by the convergence condition, contingent on the pressure change between the last two iterations.

**Fig. 3:**
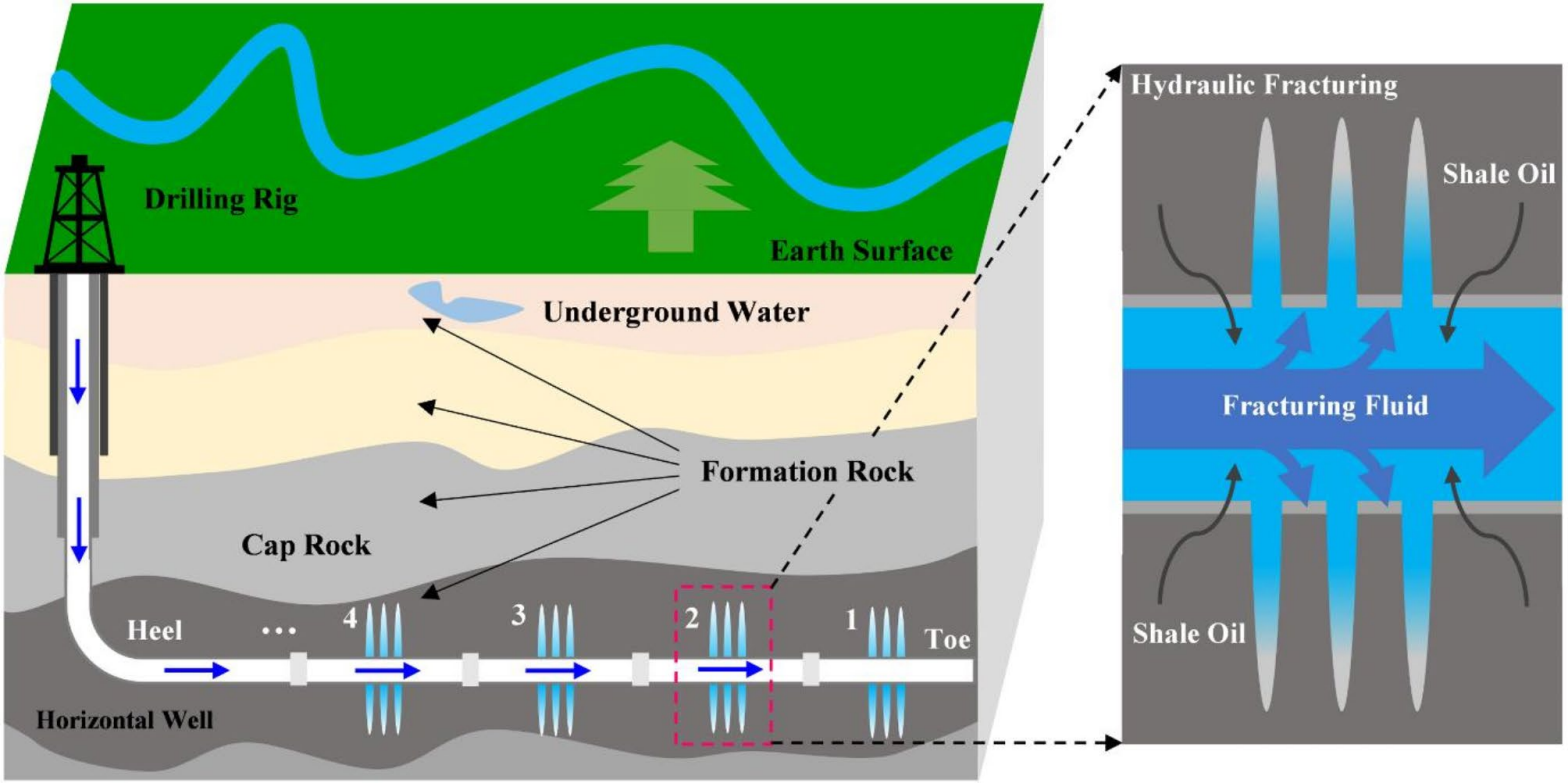
Schematic diagram of multi-stage hydraulic fracturing for horizontal well in shale reservoir.

The multi-stage hydraulic fracturing process of horizontal well in shale reservoir is depicted in Fig. 3. During the completion setting of horizontal well, the perforation location is set in each reconstruction zone. The study has 18 fracturing stages. The fracture productivity contributions are assessed through the quantitative analysis of field tracer flowback volume collected at the wellhead of the horizontal well. The wellhead sampling and tracer analysis yield a clear understanding of the productivity in each fracturing stage and establish a foundational dataset for subsequent history matching of individual stages. This method also serves as a crucial basis for the processing of stage-by-stage. Pseudo-well technology is used in the fracturing simulation with the DG model. Pseudo wells can effectively simulate the operational characteristics of actual horizontal well by advanced computer simulation^31^. The hydraulic fracturing simulation of the horizontal well in shale reservoir is finished by manipulating the perforation switch in a sequential manner.

### Numerical model

**Fig. 4:**
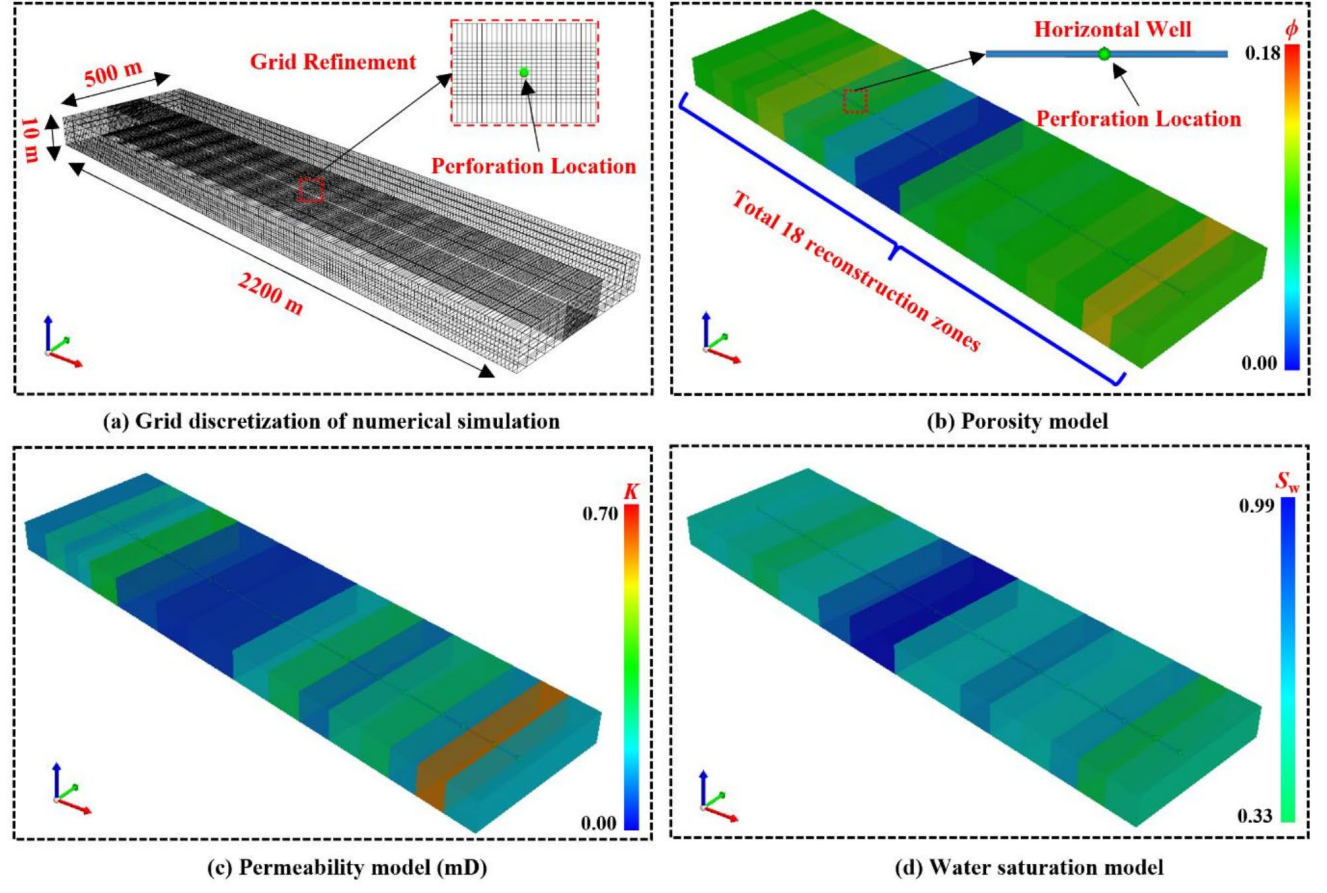
The 3D meshing DG model of shale reservoir.

Figure 4 displays the grid division results of a shale reservoir designed using CMG numerical simulation software^32^, based on realistic reservoir properties. The shale reservoir has dimensions of 2200 m × 500 m × 10 m for length, breadth, and thickness and is segmented into 220 grids in the x direction, 40 grids in the y direction, and 5 grids in the z direction. The horizontal well location in the middle layer. To accurately simulate the pressure response around the horizontal well, the grid around the horizontal well section is refined. The model exhibits heterogeneity, and the selection of model parameters is primarily based on logging data. The entire reservoir is divided into 18 reconstruction zones. According to field data, approximately 20,000 m^3^ of fracturing fluid were injected over a 10-day period during the staged multi-stage fracturing treatment. Table 1 summarizes the physical parameters of the DG model. Table 2 shows the grid setup in each reconstruction zone. The input parameters for the model are derived from actual field data. The primary variables include reservoir parameters such as porosity, permeability, water saturation, reservoir pressure, etc. as well as hydraulic fracturing and production parameters, including failure pressure, injection timing, injection volume, and production history data in the model.

**Table 1:** The DG model parameters.

Parameter	Value
Model dimensions	2200 × 500 × 10 m^3^
Reservoir depth	1945 m
Reservoir Thickness	10 m
Reservoir porosity	0.001 ~ 0.14
Reservoir permeability	0.01 ~ 0.60 mD
Initial reservoir pressure	15,800 kPa
Initial water saturation	0.33 ~ 0.99
Reservoir temperature	332 K
Oil viscosity	1.35 mPa·s
Rock compressibility	1.50 × 10^−6^ 1/kPa
Dilation compressibility	1.50 × 10^−3^ 1/kPa
Maximum allowed proportional increase in porosity	1.3
Permeability multipliers	67 ~ 170

**Table 2:** Each reconstruction zone grid setup.

Reconstruction zone	Grid setup	Reconstruction zone	Grid setup	Reconstruction zone	Grid setup
1	14 × 40 × 5	7	10 × 40 × 5	13	12 × 40 × 5
2	12 × 40 × 5	8	11 × 40 × 5	14	13 × 40 × 5
3	9 × 40 × 5	9	15 × 40 × 5	15	11 × 40 × 5
4	9 × 40 × 5	10	20 × 40 × 5	16	10 × 40 × 5
5	9 × 40 × 5	11	16 × 40 × 5	17	11 × 40 × 5
6	9 × 40 × 5	12	16 × 40 × 5	18	13 × 40 × 5

Given the actual production data of shale reservoirs, the minimal gas production is nearly negligible. This paper adopts the oil-water phase unsteady flow model to focus exclusively on the flow of water and oil in the reservoir. The isothermal seepage process is employed to simulate the flow behavior of the oil and water phases in the reservoir at each phase. The oil-water phase mass conservation equations in the flow model are presented in Eq. 3^33,34^.

3 $$\:\left\{\begin{array}{c}\frac{\partial\:}{\partial\:t}\left(\nabla\:{S}_{o}{\rho\:}_{o}\varphi\:\right)+\nabla\:\left({\rho\:}_{o}{v}_{o}\right)+{q}_{o}=0\\\:\frac{\partial\:}{\partial\:t}\left(\nabla\:{S}_{w}{\rho\:}_{w}\varphi\:\right)+\nabla\:\left({\rho\:}_{w}{v}_{w}\right)+{q}_{w}=0\end{array}\right..$$

where $\:\varphi\:$ is the porosity, $\:{q}_{\text{o}}$ and $\:{q}_{\text{w}}$ are the source and sink terms of the oil and water phases, $\:{\rho\:}_{\text{o}}$ and $\:{\rho\:}_{\text{w}}$ are the densities of the oil and water phases, $\:{S}_{\text{o}}$ and $\:{S}_{\text{w}}$ are the saturations of the oil and water phases.

Equation (4) represents the motion equations of the oil-water phase. The oil-water motion equation plays a crucial role in the flow and distribution of the oil-water phase fluid in porous media. This equation describes the flow behavior of the two-phase fluid, accounting for factors such as permeability, pressure gradient, and two-phase relative permeability to reveal the flow law of oil-water in the reservoirs.

4 $$\:\left\{\begin{array}{c}{v}_{o}=-\frac{K{K}_{ro}}{{\mu\:}_{o}}\nabla\:\left({P}_{o}+{\rho\:}_{o}gD\right)\\\:{v}_{w}=-\frac{K{K}_{rw}}{{\mu\:}_{w}}\nabla\:\left({P}_{w}+{\rho\:}_{w}gD\right)\end{array}\right..$$

where $\:K$ is the absolute permeability, $\:{K}_{\text{r}\text{o}}$ and $\:{K}_{\text{r}\text{w}}$ are the oil-phase and water-phase relative permeabilities, and $\:{\mu\:}_{\text{o}}$ and $\:{\mu\:}_{\text{o}}$ are the viscosities of the oil and water phases, $\:{P}_{\text{o}}$ and $\:{P}_{\text{w}}$ represent the pressures of the oil and water phases. And the relative permeabilities are

5 $$\:\left\{\begin{array}{c}{K}_{ro}={\left(\frac{{S}_{o}-{S}_{or}}{1-{S}_{or}-{S}_{wc}}\right)}^{2}\\\:{K}_{rw}={K}_{rwmax}{\left(\frac{{S}_{w}-{S}_{wc}}{1-{S}_{or}-{S}_{wc}}\right)}^{2}\end{array}\right..$$

where $\:K$ is the absolute permeability, $\:{K}_{\text{r}\text{o}}$ and $\:{K}_{\text{r}\text{w}}$ are the oil and water phases relative permeabilities, $\:{\mu\:}_{\text{o}}$ and $\:{\mu\:}_{\text{w}}$ are the viscosities of the oil and water phases, $\:{P}_{\text{o}}$ and $\:{P}_{\text{w}}$ represent the pressures of the oil and water phases, $\:g$ is the gravitational acceleration, $\:D$ is the depth, $\:{S}_{\text{o}\text{r}}$ is the residual oil saturation, and $\:{S}_{\text{w}\text{c}}$ is the critical water saturation.

Equation (5) is the saturation equation without considering the gas phase saturation. The saturation equation elucidates the change in the relative distribution of oil and water phases in the reservoir over time and space.

6 $$\:{S}_{o}+{S}_{w}=1.$$

Assume that the total phase velocity is

7 $$\:v={v}_{o}+{v}_{w}.$$

Neglecting the fluid compressibility and considering water saturation and oil phase pressure as the primary variables, based on Eqs. (3), (6), and (7), the following equation is derived^35^:

8 $$\:\nabla\:\bullet\:v=\stackrel{\sim}{q}\left(p,S\right)\equiv\:{\stackrel{\sim}{q}}_{w}\left(p,S\right)+{\stackrel{\sim}{q}}_{o}\left(p,S\right).$$

where $\:{\stackrel{\sim}{q}}_{w}={q}_{w}/{\mu\:}_{w}$ and $\:{\stackrel{\sim}{q}}_{o}={q}_{o}/{\mu\:}_{o}$. Combining Eqs. (4) and (7) yields the following equation:

9 $$\:v=-K[\lambda\:\left(S\right)\nabla\:p-{\lambda\:}_{w}\left(S\right)\nabla\:{p}_{c}-\left({\lambda\:}_{w}{\rho\:}_{w}+{\lambda\:}_{o}{\rho\:}_{o}\right)g\nabla\:D].$$

where $\:{\lambda\:}_{w}={K}_{rw}/{\mu\:}_{w}$ and $\:{\lambda\:}_{o}={K}_{ro}/{\mu\:}_{o}$, $\:\lambda\:$ is the total mobility, $\:\lambda\:={\lambda\:}_{w}+{\lambda\:}_{o}$, $\:{p}_{\text{c}}$ represents the pressure difference between oil phase and water phase, $\:{p}_{c}={p}_{o}-{p}_{w}$. The pressure equation can be obtained from Eqs. (8) and (9),

10 $$\:-\nabla\:\cdot\:\left(K\lambda\:\nabla\:p\right)=\stackrel{\sim}{q}-\nabla\:\cdot\:\left(K\left({\lambda\:}_{w}\nabla\:{p}_{c}+\left({\lambda\:}_{w}{\rho\:}_{w}+{\lambda\:}_{o}{\rho\:}_{o}\right)g\nabla\:D\right)\right).$$.

The oil and water phase velocities are related to the total velocity, as shown in Eq. (11) ^35^,

11 $$\:\left\{\begin{array}{c}{v}_{o}={f}_{o}v+K{\lambda\:}_{w}{f}_{o}\nabla\:{p}_{c}+K{\lambda\:}_{w}{f}_{o}({\rho\:}_{o}-{\rho\:}_{w})g\nabla\:D\\\:{v}_{w}={f}_{w}v+K{\lambda\:}_{o}{f}_{w}\nabla\:{p}_{c}+K{\lambda\:}_{o}{f}_{w}({\rho\:}_{w}-{\rho\:}_{o})g\nabla\:D\end{array}\right..$$

and introducing Eqs. (7) and (9) into Eqs. (3) and (4) to define the saturation equation,

12 $$\:\varphi\:\frac{\partial\:S}{\partial\:t}+\nabla\:\bullet\:\left\{K\right.{f}_{w}\left(S\right){\lambda\:}_{o}\left(S\right)\left(\frac{d{p}_{c}}{dS}\nabla\:S+\left({\rho\:}_{o}-{\rho\:}_{w}\right)g\nabla\:D\right)+{f}_{w}\left(S\right)\left.v\right\}={\stackrel{\sim}{q}}_{w}(p,S).$$

where $\:{f}_{\text{w}}$ and $\:{f}_{\text{o}}$ are the fractional flow function for the water and oil phases, respectively, given by $\:{f}_{w}={\lambda\:}_{w}/\lambda\:$ and $\:{f}_{o}={\lambda\:}_{o}/\lambda\:$.

This work introduces geomechanical parameters. By substituting Eqs. (1) and (2) into Eqs. (10), (11), and (12), the reservoir pressure, velocities, and saturation equations are obtained based on the DG model. These equations facilitate the characterization of the dynamic properties evolution.

A horizontal well has set up within the shale reservoir model. The fundamental objective of simulating a well is to precisely simulate the behavior of fluids drawn into the wellbore^35,36^. Well index is used to describe the productivity of horizontal wells. The calculation method of well index at the perforated zone is given by^32^

13 $$\:wi=\frac{2\pi\:\cdot\:ff\cdot\:kh\cdot\:wf}{\text{ln}\left({r}_{e}/{r}_{w}\right)+sk}.$$

where $\:ff$ is the form factor, $\:wf$ is the well fraction, and $\:ff$ and $\:wf$ specify the well geometry parameters, $\:kh$ is the length of a grid in *k* direction, $\:sk$ is the skin factor.

The theoretical framework of rock deformation and the numerical simulation methodology are elucidated in this section. Considering flow space, multiphase theory, and thermodynamic principles, the fracturing production process of horizontal wells in shale reservoirs is accurately reconstructed using pseudo-well numerical characterization technology coupled with the DG model. On the basis of the numerical model mentioned above, a high-precision simulation workflow for hydraulic fracturing of horizontal wells in shale reservoirs is proposed, which aims to accurately capture the complex dynamic behaviors during hydraulic fracturing and production.

## Results and discussion

This study enhances understanding of the underlying processes and provides valuable scientific guidance for analyzing the dynamic evolution of the reconstruction zones.

### History matching

History matching in reservoir numerical simulation is a key engineering practice for achieving optimally matched results. This approach primarily aims to examine the numerical simulation’s accuracy and reliability by comparing it with actual reservoir behaviors.

#### Multi-stage history matching

The shale reservoir is subjected to 18 successive hydraulic fracturing stages. History matching of reservoir production employs a stage-by-stage approach, ultimately achieving the history matching objective for the entire horizontal well production. Practical field practices only provide aggregated production data for the entire horizontal well. To attain this objective, the production data must be segmented. The segmented data is matched to each fracture reconstruction zone using the results of field tracer segmented productivity test. The segmented serve as the foundation for each stage of history matching. Table 3 provides statistics on oil and water flowback volume from the fracturing operations in stage 1. This data is crucial for determining the allocation of oil and water production for each reconstruction zone.

**Table 3:** The major operation and flowback parameters of the fracturing stage 1.

Parameter	Value
Failure pressure	48.2 MPa
Injection volume	790.5 m^3^
Water flowback volume	13.75 m^3^
Oil flowback volume	3.45 m^3^

**Fig. 5:**
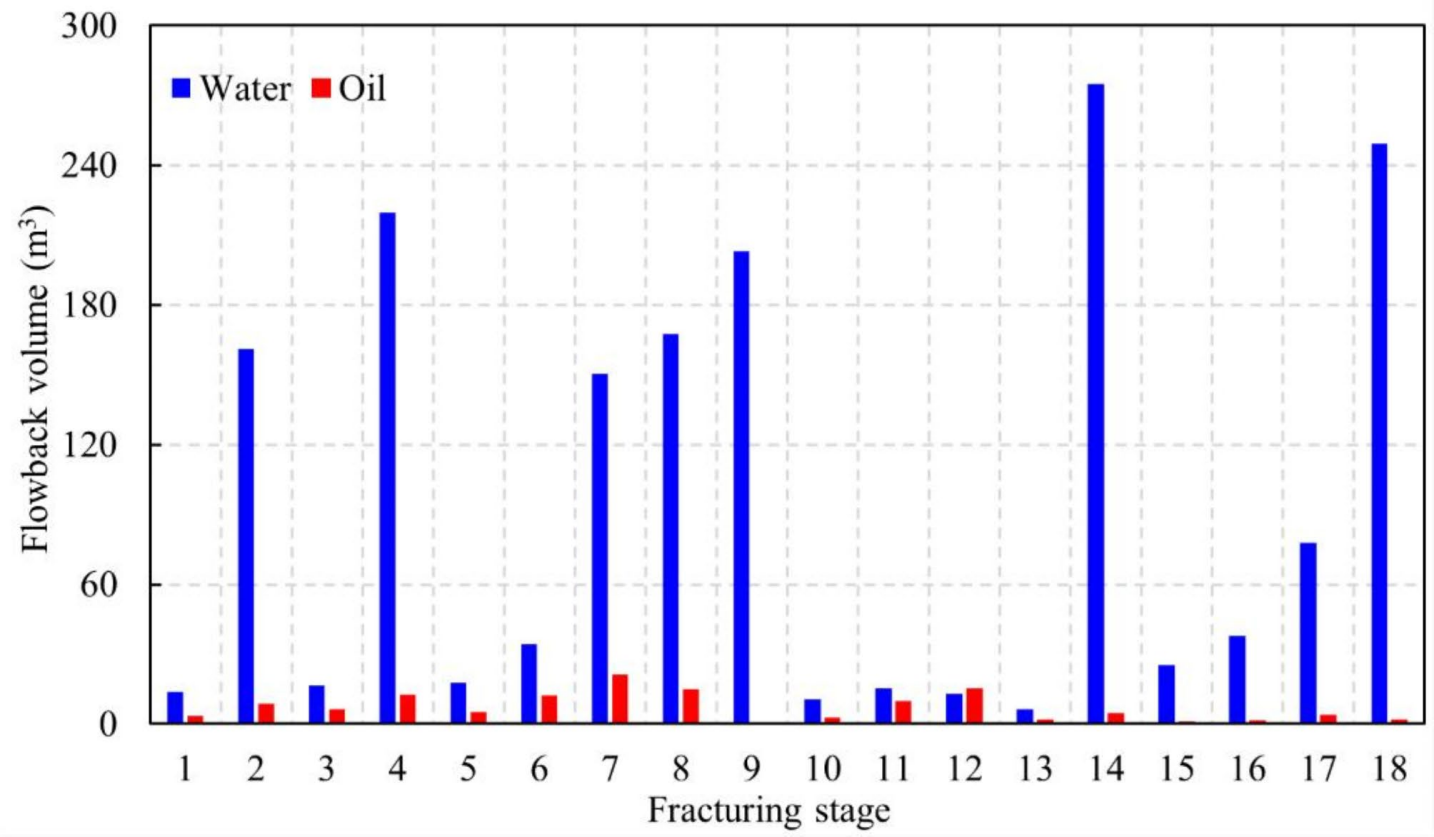
Oil-water flowback volume in each fracturing stage.

Historical oil and water production data for fracturing stage 1 are derived from the single-stage flowback volume depicted in Fig. 5. The water flowback volume for stage 1 amount to 13.75 m^3^, constituting 0.81% of the total water flowback volume, while the oil flowback volume is 3.45 m^3^, comprising 2.70% of the total oil flowback volume. Production is allocated to each stage of hydraulic fracturing according to the percentage of oil and water output from each reconstruction. This ensures that production distribution across each reconstruction zone is handled logically and precisely.

**Fig. 6:**
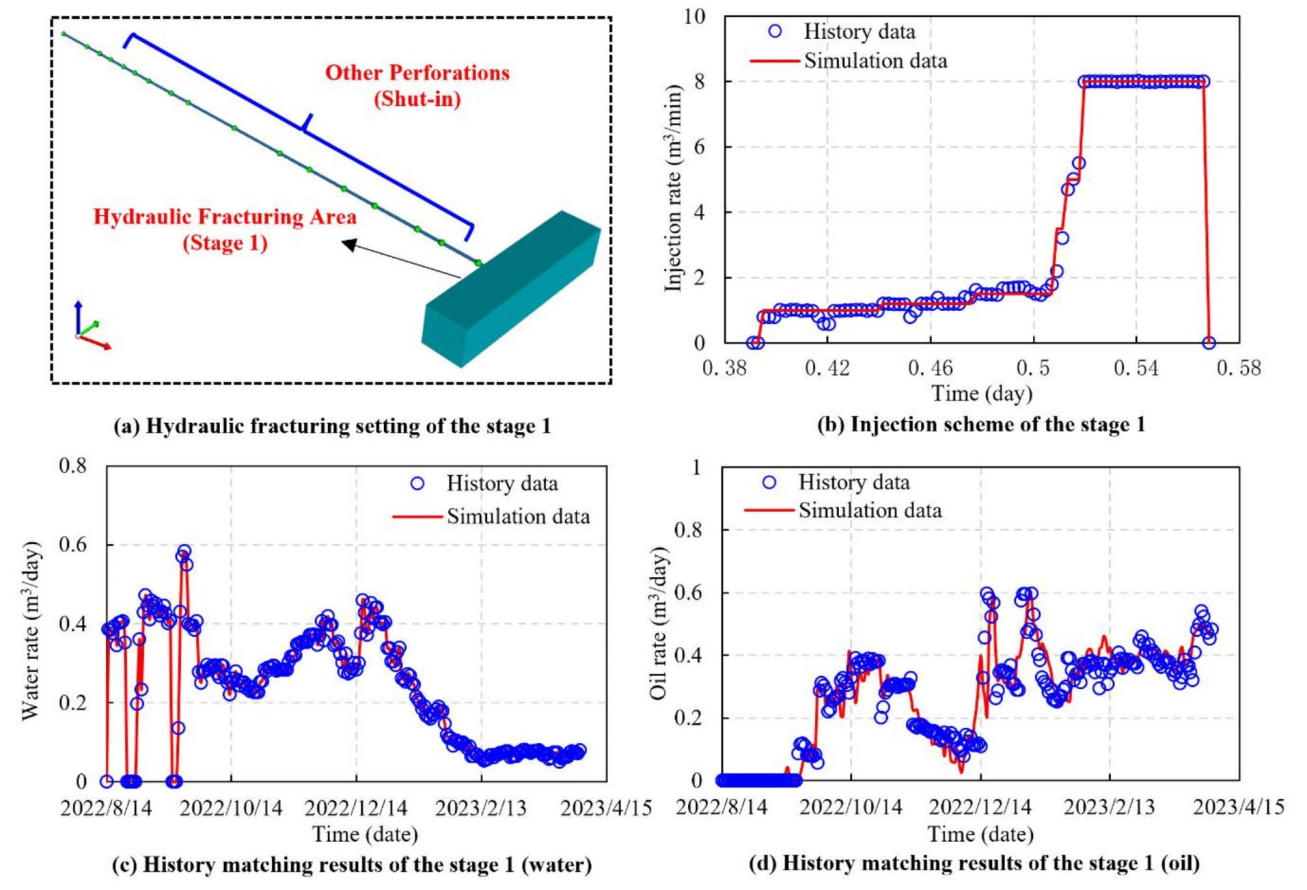
Multi-stage injection scheme and history matching results (the fracturing stage 1).

The history matching of a single hydraulic fracturing stage is shown in Fig. 6. Hydraulic fracturing is carried out on the first stage when other perforations are shut-in (Fig. 6a). Figure 6b depicts the injection scheme for the first fracturing stage controlled by the injection switch. The injection rate begins at a low level, then maintains at a higher injection rate and stops once the predetermined injection volume has been achieved. The application of multi-stage history matching for stage 1 obtains satisfactory performance. A successful history matching of the stages contributes to optimizing the DG model and improves the overall matching accuracy.

#### Full-hole history matching

**Fig. 7:**
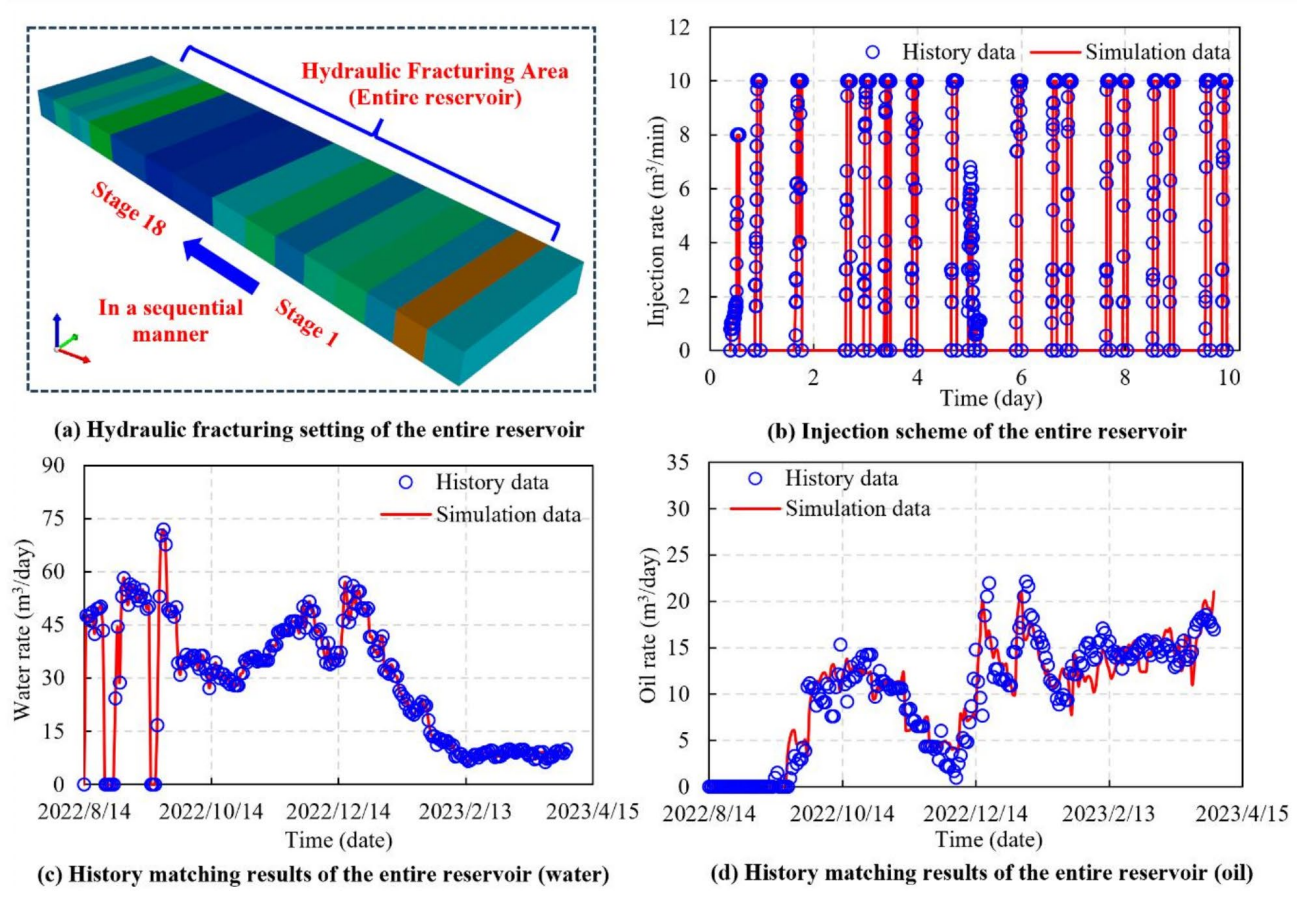
Full-hole injection scheme and history matching results (the entire reservoir).

In order to ensure that the injection process in-depth simulates the temporal sequence and injection volume of the actual field practice, the fracturing fluid injection scheme is designed to be consistent with the field injection data. Staged simulation results are used to match the history of fracturing and production in the entire reservoir. The full-hole hydraulic fracturing process is carried out from the fracturing stage 1 to the fracturing stage 18 by the perforation point switches in a sequential manner (Fig. 7a). Over the course of a 10-day fracturing operation, 18 fracturing stages are executed. The injection scheme is illustrated in Fig. 7b. Achieving the full-hole history matching involves migrating and integrating variable parameters associated with the multi-stage history matching.

The results of the history matching of the daily production from horizontal well are shown in Fig. 7c and d. Circles in the figures represent the actual data on daily water and daily oil production during the production phase, while lines illustrate the numerical simulation results from the DG model. The figures illustrate the degree of the matching between the simulation results and the actual historical production. And the numerical simulation results of the DG model closely resemble the actual well production data. The key factors for history matching are the parameter values of the dilation-recompaction model, as listed in Table 1. In parallel, one of the most important ways for adjusting the DG model production trend is to modify the relative permeability curve throughout the simulation. Achieving satisfactory history matching allows for enhancing predictive performance and optimizing the model, and provides more dependable data for production planning and decision-making in the future.

### Evolution of reservoir reconstruction zone

Generally, a high-pressure liquid is pumped into the reservoir rock, causing in to dilate and subsequently fracture. The formation of these fractures increases the permeability of the reservoir and promotes fluid migration. Hydraulic fracturing affects the orientation, length, and connectivity of fracture. The enhanced fracture permeability is thought to jointly impact both the matrix and the fractured rock. The DG model treats resulting fractures as a continuum^28^, which implies that fractures interact with the matrix to collectively affect permeability, rather than acting as isolated zones of fragmentation.

#### Reservoir reconstruction scale

**Fig. 8:**
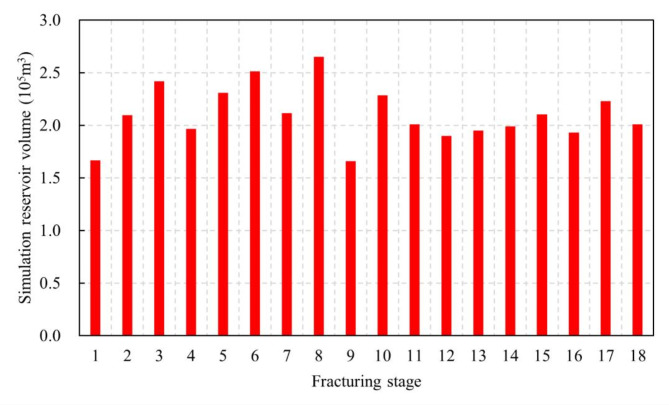
Effective stimulated reservoir volume in different reconstruction zones.

Field cases in shale reservoirs demonstrates that the effective stimulated reservoir volume (ESRV) is a crucial factor influencing the production of horizontal well subjected to hydraulic fracturing in shale reservoirs. Accurate characterization of the ESRV is pivotal in evaluating shale reservoir fracturing operations and predicting the productivity of horizontal wells. Figure 8 presents the statistical data of ESRV generated by each reconstruction zone following the fracturing operation. The ESRV of each reconstruction zone varies due to differences in operational parameters and reservoir physical properties.

**Fig. 9:**
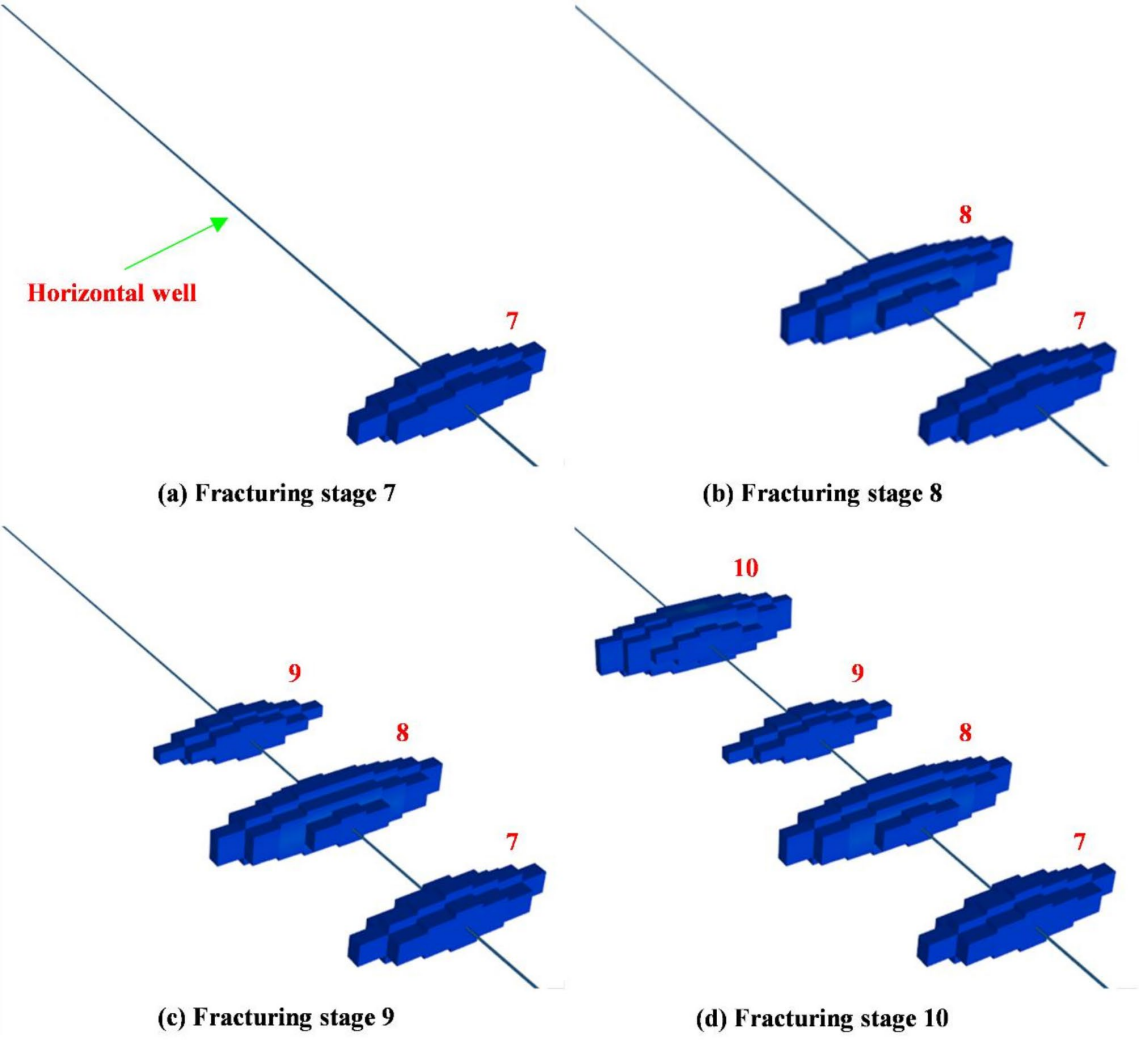
A 3D reconstruction zone on fracturing stage 7, 8, 9 and 10.

The hydraulic fracturing reconstruction scale for stages 7, 8, 9, and 10 are provided a three-dimensional visualization in Fig. 9. Notably, the reconstruction scale of stage 8 exceeds that of the other stages, attributed to specific fracturing operation and reservoir parameters in this stage.

**Fig. 10:**
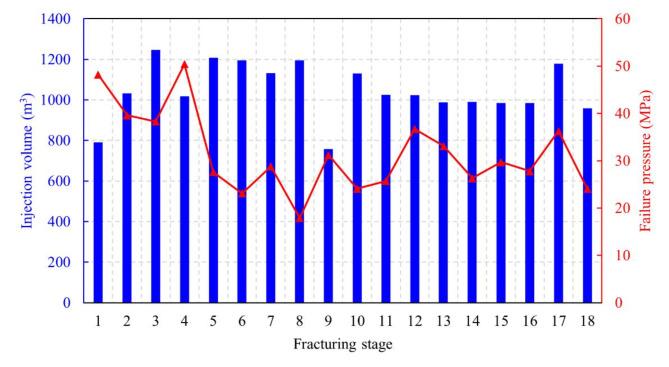
The amount of fracturing fluid and rock failure pressure in different fracturing zones.

The fracturing operation parameters significantly influence the ESRV including both the injection volume and the failure pressure of formation rock. Figure 10 illustrates the rock failure pressure and the amount of fracturing fluid injected in each reconstruction zone. The histogram represents the fracturing fluid injection volume, while the line chart represents the failure pressure of each reconstruction zone.

**Fig. 11:**
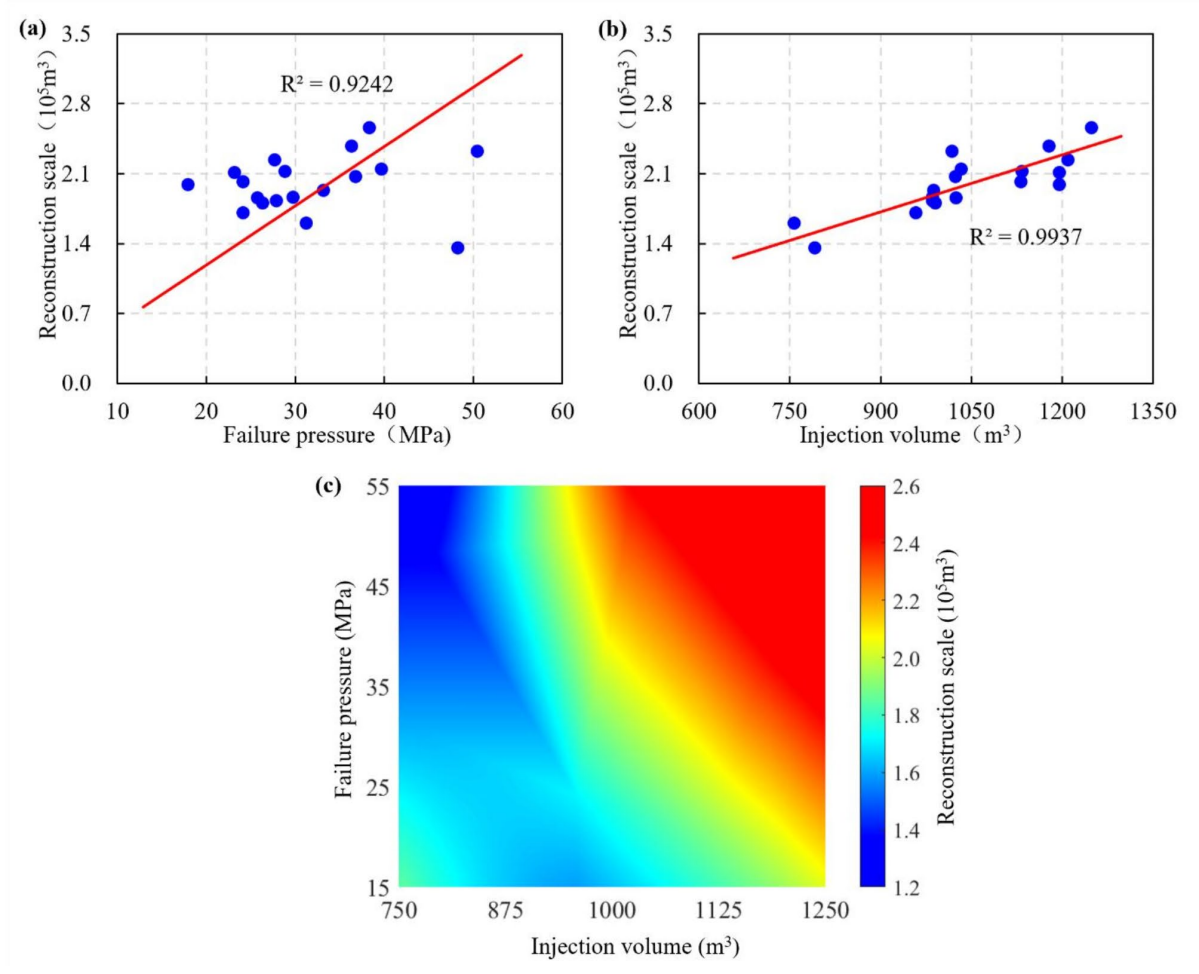
Correlation of failure pressure and injection volume with reservoir reconstruction scale. (a) One-factor analysis for failure pressure, (b) One-factor analysis for injection volume, (c) Multi-factor heatmap analysis^37^.

The correlation between failure pressure and injection volume with the reservoir reconstruction scale is shown in Fig. 11. Combined with the one-factor analysis and the heatmap uncovering the influence of multi-factor interactions, from the direction of the diagonal of the heatmap, there is a positive correlation between the failure pressure and injection volume with the reservoir reconstruction scale. Despite the high failure pressure and the delayed onset of the dilation state in rock, with the increase of the injection volume, the delayed dilation state will be offset. Meanwhile, with a certain amount of injected fracturing fluid, the pressure head advances efficiently with minimal energy loss when the reservoir exhibits favorable physical properties, thereby generating a larger reconstruction scale. However, with high failure pressure, insufficient injection volume will lead to poor stimulation. As to specific reservoirs with certain failure pressure, the meticulous design and the control of fracturing volume are paramount to ensure the hydraulic fracturing performance of horizontal wells in shale reservoirs. Excessive or insufficient stimulated reservoir volume may impact the effective production of horizontal wells. Thus, the optimal stimulated reservoir volume must take into account the reservoir properties, the geological conditions, and the fracturing fluid injection volume.

#### Evolution of reservoir properties

**Fig. 12:**
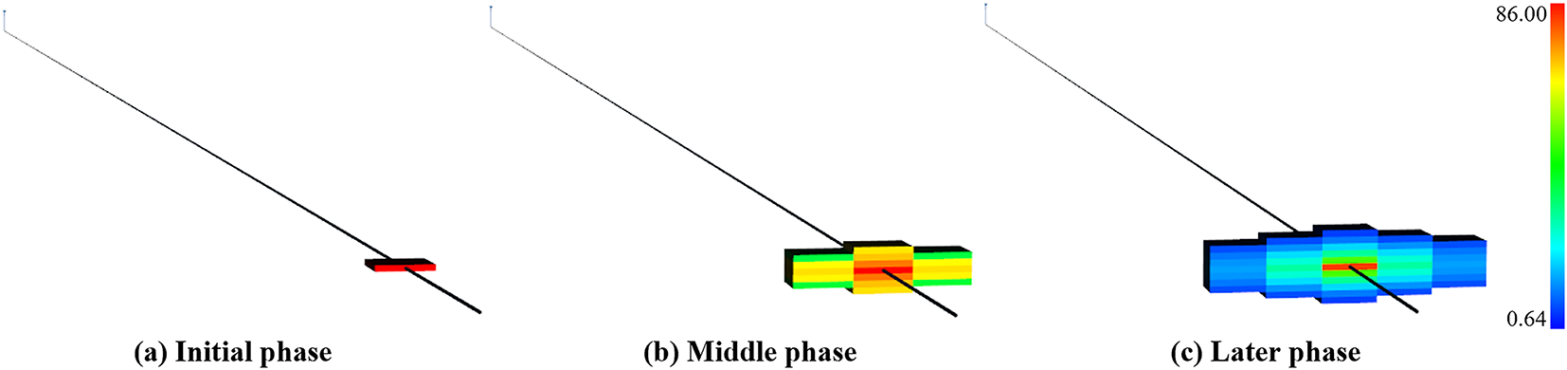
A 3D evolution of permeability (mD) in the first fracturing stage.

The time series evolution for fracture zone permeability before and after hydraulic fracturing is intuitively shown in Fig. 12. Within a brief timeframe, the substantial injection of fracturing fluid will generate high pressure at the perforation location. The rock will dilate and fracture upon reaching the rock failure pressure. The initial permeability at this stage is merely 0.16 mD. Nevertheless, the volume of injected fracturing fluid amounts to 790.5 m^3^ during the fracturing operation, and the maximum apparent permeability in this reconstruction stage reaches as high as 86 mD.

**Fig. 13:**
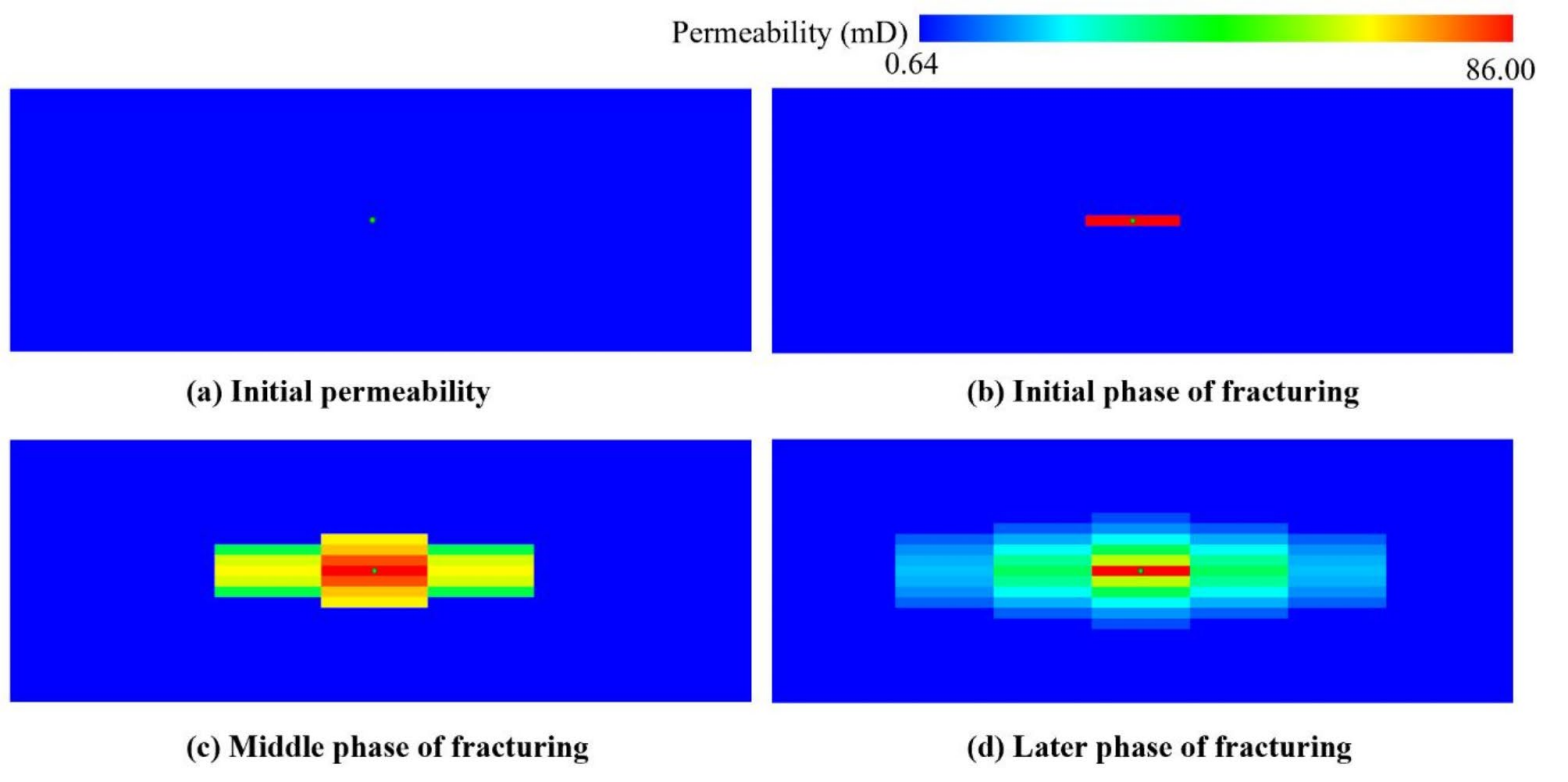
Evolution of permeability (mD) in the first fracturing stage.

Hydraulic fracturing markedly enhances the effective permeability of reservoirs. However, an understanding of the dynamic performance of reservoirs in terms of permeability evolution over time is necessary. The time series evolution of permeability around the perforation location during the hydraulic fracturing operation has been demonstrated in Fig. 13. The evolution in permeability exhibits a rugby ball shape, corresponding to the alteration in three-dimensional shape in Fig. 12. With the injection of high-pressure fracturing fluid, the permeability increased significantly. The fracture half-length of the fracture zone reaches 148 m, which basically meets the design requirements.

**Fig. 14:**
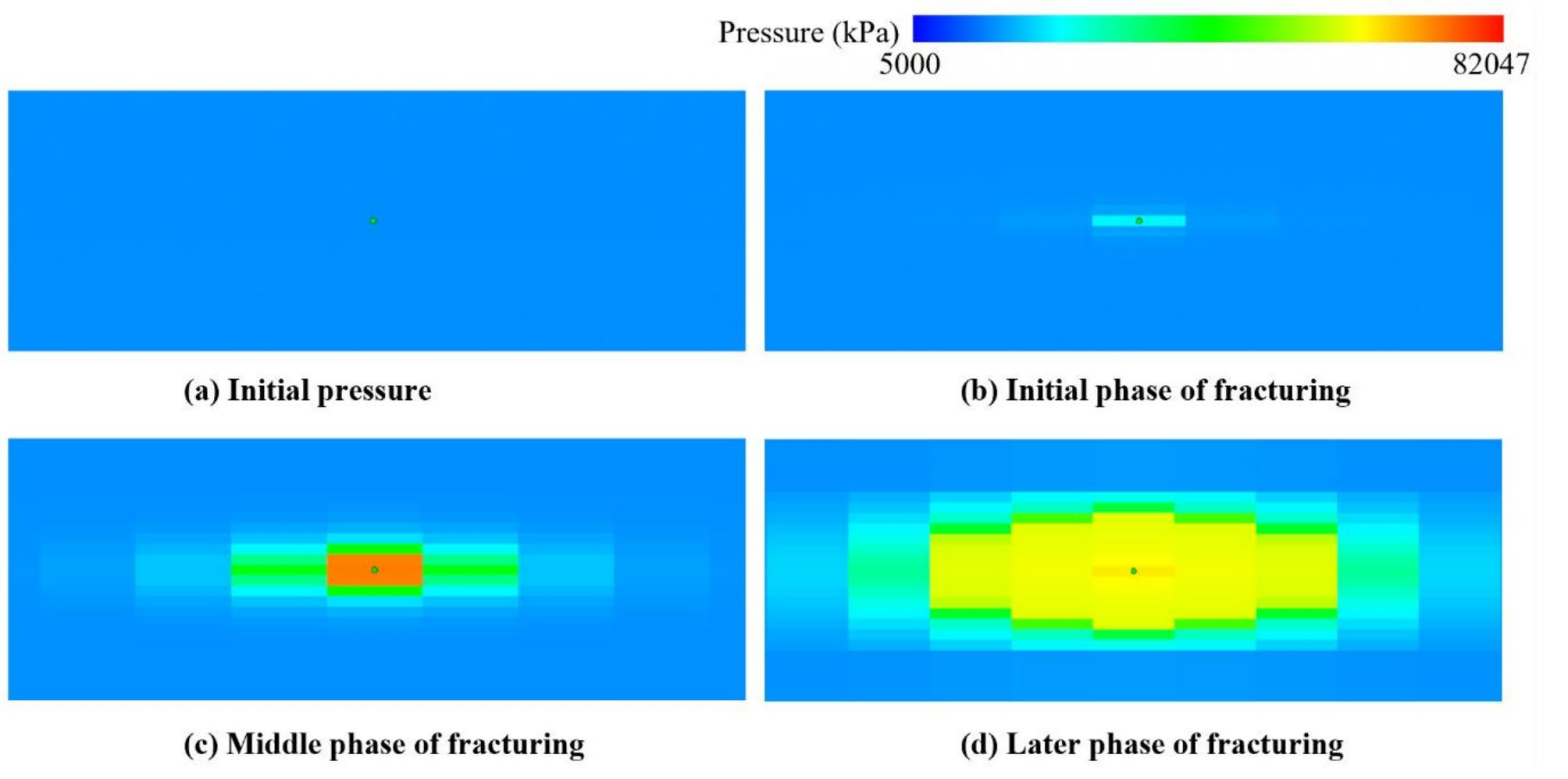
Evolution of formation pressure (kPa) in the first fracturing stage.

The time series pressure evolution is significant visible in Fig. 14, which depicts the process of pressure change during the injection of fracturing fluid. In the initial phase, the pressure rises rapidly to reach rock failure pressure, and fractures begin to form in the reservoir under the action of hydrodynamics and geomechanics. Then the pressure gradually rises to its maximum level while the high-pressure fluid pushes the fractures outwards. The pressure response in the whole process reflects the dynamic evolution of fracture formation and propagation.

**Fig. 15:**
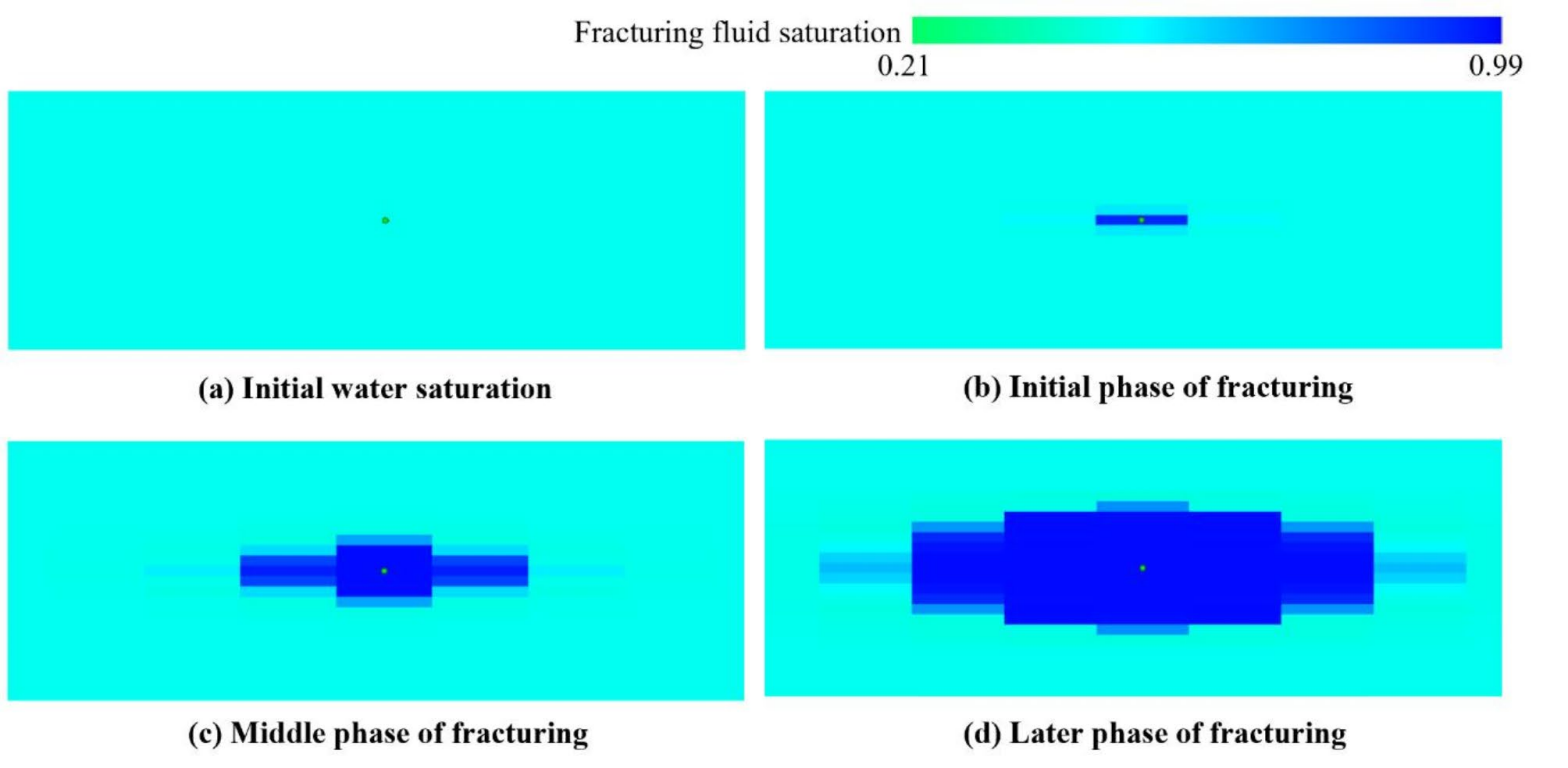
Evolution of fracturing fluid saturation distribution in the first fracturing stage.

Figure 15 shows the diffusion process of fracturing fluid in the fracture zone. It is also extremely important to clarify the fracturing fluid distribution in hydraulic fracturing operation. The continual high-pressure fluid is injected quickly diffuses and fills the matrix pores. The fracture network expands and progressively creates a new high-permeability channel. The evolution pattern of reservoir permeability, pressure, and fracturing fluid distribution in the reservoir fracture zone are all in accord during the whole hydraulic fracturing process.

**Fig. 16:**
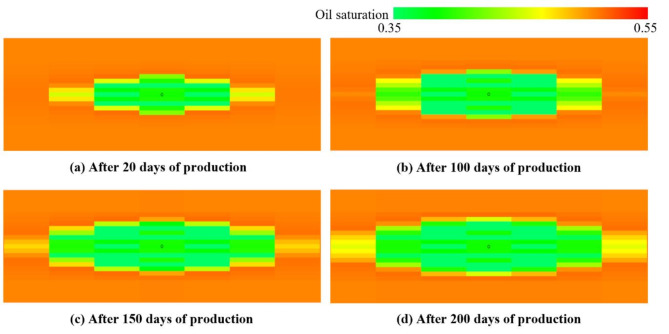
Evolution of oil saturation in production phase.

The distribution of oil saturation around the horizontal wellbore is shown in Fig. 16. After the horizontal well has been shut-in for a period, the production phase begins. The oil saturation in the vicinity of the horizontal well is notably lower than in other parts of the reconstruction zone in early production phase. This is primarily attributed to the filling of fracturing fluid in the fracture zone. The crude oil in the matrix progressively migrates to the high-permeability area surrounding the wellbore during the shut-in period. With the progression of the production, the oil saturation displays an expanding decrease in the fracture zone outward, as seen in Fig. 16. It is noteworthy that the permeability of the high-permeability area remains at the level of 46 mD during the production phase. The numerical simulation results demonstrate the DG model can effectively simulate the complex process of fracturing operation and production.

## Conclusions

This work develops the DG model to describe the process of horizontal well hydraulic fracturing operation and production in shale reservoirs. The actual field data is effectively applied to the model and workflow. This work is of vital importance for multi-stage history matching and dynamic properties evolution. Some key conclusions are drawn as follows:

1. This study presents the DG model focusing on the coupled the unsteady seepage model of multi-stage fractured horizontal wells and the dilation-recompaction model to characterize the hydraulic fracturing operation and production in shale reservoirs. The DG model is established using the data that is designed to be consistent with the actual field.
2. The numerical simulation process comprehensively accounts for the interaction of various phases, including high-pressure fluid injection, fracture formation and expansion, and production. Utilizing logging, production tests, and other relevant data, the construction procedure aligns closely with field practice, and the geological model of the shale reservoir is systematically established through the segmentation of the data. One of the key factors in the accurate modeling of the hydraulic fracturing dynamic operation lies in the application of the shale reservoir geomechanical parameters.
3. Multi-stage history matching is performed to reconstruct the distribution relationship between each reconstruction zone and the overall productivity. The numerical simulation results of the DG model closely resemble the actual well production data. It adeptly characterizes the distribution of fracturing fluid in the shale reservoir, the geometry of fractures, and the evolution of permeability, pressure, and oil saturation.
4. An integrated process of fracturing production is established to achieve a comprehensive and in-depth analysis of the entire reservoir from the fracturing operation in the shale reservoir to the seamless transition of the production phase. Optimizing the design of hydraulic fracturing, increasing the horizontal well production, and making the best use of reservoir resources are all made possible by the simulation technology.

## Data Availability

Data will be made available on request. Please contact Prof. Zhan via zhanjie_petro@163.com.
